# SwinConvNeXt: a fused deep learning architecture for Real-time garbage image classification

**DOI:** 10.1038/s41598-025-91302-7

**Published:** 2025-03-07

**Authors:** B. Madhavi, Mohan Mahanty, Chia-Chen Lin, B. Omkar Lakshmi Jagan, Hari Mohan Rai, Saurabh Agarwal, Neha Agarwal

**Affiliations:** 1 Department of Computer Science and Engineering, Vignan’s Institute of Information Technology, Duvvada, Visakhapatnam, Andhra Pradesh India; 2 Department of Computer Science and Information Engineering, National Chin-Yi University of Technology, Taichung, 411 Taiwan; 3 School of Computing, Gachon University, 1342 Seongnam-daero, Sujeong-gu, Seongnam-si, Gyeonggi-do 13120 Republic of Korea; 4 School of Computer Science and Engineering, Yeungnam University, Gyeongsan, 38541 Republic of Korea; 5 School of Chemical Engineering, Yeungnam University, Gyeongsan, 38541 Republic of Korea

**Keywords:** Waste segregation, Deep learning, Swin transformer, ConvNeXt, Computer vision, Spatial attention mechanism, Computer science, Information technology

## Abstract

Waste management handles all kinds of waste, including household, industrial, municipal, organic, biomedical, biological, and radioactive wastes. People still face challenges in proper disposal methods for different types of waste, including landfill-bound items, recyclable materials, and biodegradable waste. Inadequate waste management poses a significant and multifaceted global challenge. The conventional method of segregating waste is a time-consuming and ineffective method that wastes human power and money. To address this issue in real time, sophisticated and sustainable waste management systems need to be implemented. The latest advancements in computer vision and deep learning offer efficient solutions for effective recycling and waste management. Existing deep learning models exhibited various limitations, such as detection accuracy and computational inefficiency, particularly when dealing with objects of varying sizes and exhibiting high degrees of visual similarity. These limitations generate various challenges in effectively capturing and representing the nuanced features of visually similar objects. To address this problem, we proposed the stacking of an enhanced Swin Transformer, improved ConvNeXt, and a spatial attention mechanism. The enhanced Swin transformers incorporate two key components- hierarchical feature extraction and shifting window mechanism to extract the global features from the garbage images effectively. The shifting window mechanism extracts the most important features from various regions of the images to identify the objects. In contrast, the hierarchical feature extraction captures long-range dependencies within the image to effectively identify different types of garbage. The improved ConvNext block with optimized parameterization extracts the local features of the image. This enhanced feature extraction capability enables the model to effectively discern fine-grained details of individual garbage particles, such as shape, texture, and subtle variations in color and appearance, leading to more accurate classification results. When we evaluated the performance of the proposed model using the publicly available Garbage Classification dataset, it attained 98.97% accuracy, 98.42% Precision, and 98.61% Recall. Due to its lightweight and low computational time and power, the proposed model surpasses the existing state-of-the-art deep learning models.

**Supplementary Information:**

The online version contains supplementary material available at 10.1038/s41598-025-91302-7.

## Introduction

Waste can be solid, liquid, or gaseous, and each one needs a particular approach to handling and disposal. Waste may consistently be dangerous to human health. In addition, there are health risks associated with managing solid waste. Human activities produce waste when crude substances are extracted and refined. The goal of waste management is to reduce the negative consequences that waste has on the atmosphere, global resources, people’s health, and aesthetics^1^. Decreasing the adverse effects of garbage on the ecosystem and human health is the objective of waste management. Effective waste management, particularly handling Municipal Solid Waste (MSW) from households, businesses, and industries, is crucial for sustainable and livable communities. In 2018, the EPA reported that a total of 292.4 million tons of MSW were generated in the United States. From the total generated waste, only 69 million tons were recycled, 25 million tons were decomposed, and 94 million tons were reformed^2^.

From the current statistics released by the World Bank^3^, planetary waste will rise by 70% without urgent intervention by 2050. In order to achieve effective waste management, there is a need to integrate technological advancement and economic viability with sustainable needs. The main strategies were technical disposal facilities and the promotion of environmentally friendly products. Through this, it is possible to get considerable environmental benefits in terms of quality air and water, reduced greenhouse gas emissions, and preservation of natural resources. Along with the environmental benefits, proper management of waste will improve public health by minimizing exposure to hazardous substances and preventing the spread of diseases.

The recycling component, in particular, distinguishes waste management. Because recycling rubbish reduces the need to cut down trees, recycling may help us decrease the volume of energy we use and also the resources we use on Earth. When waste management is done correctly, it not only removes surrounding waste but also reduces the potency of greenhouse gases obtained from the collected waste, such as carbon monoxide and methane. The depth of the current landfills and incineration will be reduced, reducing the detrimental elements that impact the surroundings. Every phase of the process, from the first phase of collecting to the last phase of segregation, needs labor, which ultimately results in the creation of numerous job possibilities. With a cost of $50 for every ton of garbage that continues to be put in landfills, recycling it in the right way costs $150 per ton, which is why most businesses prefer to use the landfill approach instead.

**Fig. 1:**
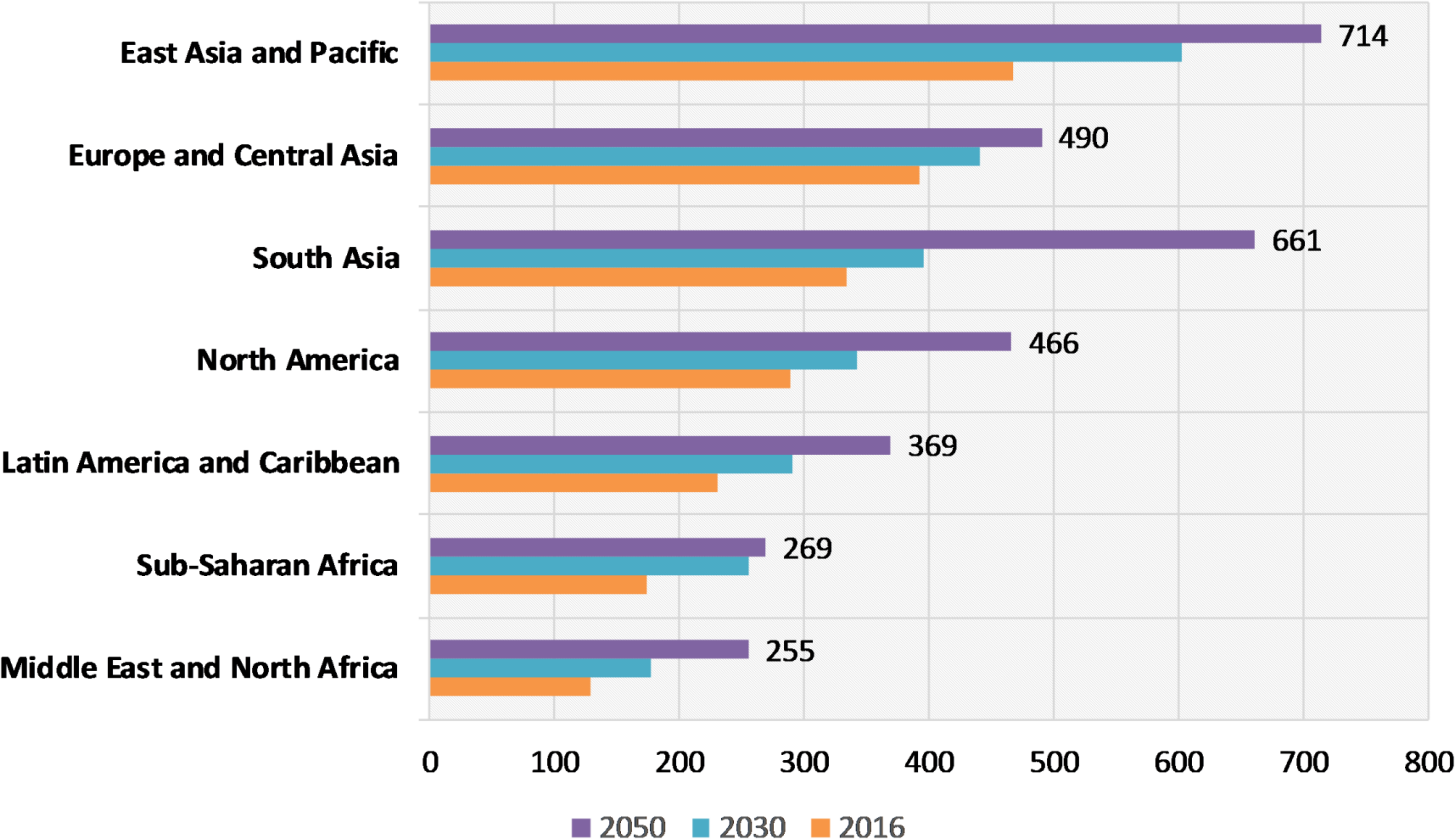
Projected Annual Regional Waste Generation^4^.

Although environment friendly, the recycled product is anticipated to have a shorter lifespan than the original one. However, many of these waste management approaches are only utilized on a limited scale, are basically restricted to residences, businesses, and educational institutions, and are not frequently applied in large-scale businesses and global companies. The Statistics of waste produced throughout the world reported by the World Bank are shown below in Fig. 1.

The escalating global waste crisis necessitates innovative and efficient waste management solutions. Traditional manual waste classification methods are time-consuming, manual labor-intensive, and prone to human error. This research is motivated by the urgent need to develop an automated and intelligent system for real-time garbage classification. By leveraging the power of Artificial Intelligence, we aim to create a Deep learning (DL) based system that can accurately and efficiently classify various types of garbage, such as paper, plastics, glass, and organic waste. This system significantly improves efficiency in waste management, reduces environmental pollution, and promotes sustainable waste disposal practices.

Deep Learning, a subfield of Machine Learning (ML), offers a powerful computational methodology to revolutionize waste management practices. By employing the most optimized algorithms and leveraging large datasets to minimize detection errors, recent advancements in DL ultimately contribute to a more sustainable and efficient waste management system.

This research aims to develop a novel DL model for efficient and accurate garbage classification. To achieve this goal, the following objectives will be addressed:

- Proposed a novel architecture that combines the strengths of Swin Transformer, ConvNeXt, and spatial attention mechanism.
- Design and implement a lightweight deep learning architecture that minimizes computational cost while maintaining high classification accuracy.
- Demonstrate superior performance in terms of accuracy, efficiency, and robustness on a challenging garbage classification dataset.
- Conduct thorough experimental analysis to evaluate the model’s performance and compare it with state-of-the-art methods.

The remaining part of the research paper is arranged as follows: Section “Literature survey” describes the Literature Survey. Section “Deep learning” deals with Deep Learning. Section “Proposed method” presents the Proposed method. Section “Experimental results and analysis” provides experimental results and analysis. Finally, the last portion of the paper concludes the research in Section “Conclusion and future scope”.

## Literature survey

Several initiatives have been undertaken in recent years to minimize the impact of improper waste disposal. Garbage image classification research studies have been conducted using neural networks and DL-based models. Different research papers have employed a variety of approaches to address this problem. Each approach is unique and utilizes its methods to achieve high accuracy. Lilhore et al.^5^ proposed a novel hybrid model that integrates Convolutional Neural Networks (CNNs) with Bidirectional Long Short-Term Memory (Bi-LSTM) for accurate and efficient garbage classification. By leveraging transfer learning with prominent CNN architectures (VGG-16, VGG-19, ResNet-34, ResNet-50, AlexNet) and incorporating Bi-LSTM for temporal dependencies, the proposed model achieves a classification accuracy of 96.78%, surpassing existing methods by a significant margin. This enhanced performance demonstrates the model’s potential for improving waste management practices by facilitating accurate and efficient waste segregation, ultimately contributing to a more sustainable and environmentally friendly future. Paneru et al.^6^ proposed a unique DL model for garbage classification. In this paper, a unique PyTorch model linked with a Tkinter-based Recycling Recommendation Application deals with the significant problem of managing waste. The proposed model shows a remarkable accuracy of 96% on the validation dataset. Combining advanced machine learning and user-centered design (UI) is a big step toward greener garbage disposal methods and more effective waste management. Dalal et al.^7^ introduce a novel framework integrating Convolutional Neural Networks (CNNs) with Bidirectional Long Short-Term Memory (BiLSTMs) to enhance efficiency and sustainability. CNNs analyze spatial relationships, optimize resources, and identify patterns, while BiLSTMs capture temporal dependencies for accurate demand forecasting. This hybrid approach enables real-time adaptability and improved resilience. Predictive analytics optimize inventory, minimize stockouts, and reduce lead times. Furthermore, the framework promotes sustainability by optimizing transportation routes, minimizing carbon emissions, and facilitating environmentally conscious sourcing. The model demonstrates strong performance, indicating its potential to enhance the efficiency and sustainability of modern supply chains significantly. Lilhore et al.^8^ proposed a novel hybrid deep learning model for accurate waste classification, combining Convolutional Neural Networks (CNNs) with Long Short-Term Memory (LSTMs) and incorporating transfer learning techniques. The model leverages pre-trained CNN architectures (VGG-16, ResNet-34, ResNet-50, AlexNet) and an improved data augmentation approach to address overfitting and data imbalance. Experimental results on the TrashNet dataset demonstrate the superior performance of the proposed model compared to existing methods, achieving a high precision of 95.45% with the Adam optimizer, highlighting its potential for enhancing waste management practices and promoting environmental sustainability.

Mineral extraction plays a crucial role in the global economy, yet unsustainable practices pose significant environmental and social challenges. This research explores the potential of deep learning-enabled FinTech solutions to enhance mineral identification and promote sustainable resource extraction. Authors proposed a quantized EfficientDet model^9^ for real-time mineral identification during mining operations. This approach leverages post-training quantization to optimize model size and computational cost while maintaining high accuracy. Our findings demonstrate that the quantized model achieves superior performance compared to the original floating-point model, paving the way for more efficient and sustainable mineral exploration and extraction. Furthermore, the integration of FinTech technologies enables the development of innovative financial models that assess environmental and social risks, guiding investors toward more responsible and sustainable mining practices. Mohammed Imran Basheer Ahmed et al. published a paper in 2023. This paper presents a thorough examination of waste categorization utilizing pre-trained models MobileNetV2, ResNet50V2, and DenseNet169 in addition to advanced CV (Computer Vision) algorithms like CNN’s (Convolutional Neural Networks)^10^. The study’s conclusion showed that the CNN model attains 88.52% accuracy on the Garbage classification dataset. Meanwhile, the existing models MobileNetV2, DenseNet169, and ResNet50V2 had accuracies of 98.95%, 97.60%, and 94.40%, respectively, on the same dataset. These outcomes are remarkable compared to the progressive studies in the literature. The work has the potential to automate garbage classification, facilitate efficient waste management, and contribute to a more livable and environmentally friendly future. Srilatha et al. create convolution feature maps using a base network, ResNet 101. During the first stage, these convolution features are used to build a Region Proposal Network (RPN)^11^, which generates bounding rectangles, objectness scores, and 256-dimensional feature vectors for various anchor boxes. Cardboard, glass, metal, paper, and plastic are the five categories of solid waste that can be classified and localized in the next step when a SoftMax layer and regressor are trained using the region recommendations. The suggested Faster RCNN applies RPN, a tiny neural network overlying the final convolution layer’s feature map, anticipating object existence and also bounding boxes. The Faster RCNN, along with ResNet 101 and RPN, attained an accuracy of 96.7% on the Garbage Classification dataset according to experimental results. Vijayalakshmi et al. describe the EBMOHDL-WC^12^ model under the Internet of Things (IoT) empowered sustainable environment. The IoT devices can continue the data collection process according to the EBMOHDL-WC system that has been presented. The proposed technique then employs the MobileNetv2 model to extract all features, and the hyperparameter modifications of the MobileNetv2 technique were executed by the EBMO technique, presenting the work’s uniqueness. The proposed EBMOHDL-WC attains an accuracy of 98.04%, Precision of 93.89, Recall of 93.33, and F-Score of 93.60 over the Garbage Classification dataset.

Bobde et al. used the EfficientNet-B3 CV model^13^, which has a more efficient design and performs similarly to other deep convolutional neural networks. The researchers concluded that the best tool for classifying waste would be computer vision. The Computer Vision model will receive the image as test data. Then, the model will use it to execute its forecasts and determine which category the waste belongs to. Subsequently, everything should be sorted into the appropriate waste containers to achieve better performance. The proposed model achieves 97% accuracy on the Garbage classification dataset. Tatke et al. evaluate and contrast a number of different techniques and strategies^14^, including Simple CNN, ResNet50, and VGG16. The related analysis and research demonstrate the effectiveness of using all methods. The authors claimed that the proposed ResNet50 performs exceptionally well, and it attains an accuracy of 95.93% on the Garbage Classification dataset. Furthermore, the VGG16 network offers excellent performance, which fulfills requirements for regular usage, and it attains 75.6% accuracy. Researchers also used a Histogram of Oriented Gradients (HOG) along with an SVM classifier, which is a feature descriptor that essentially finds the pertinent information from the image that can help the classifier enhance prediction, and it attains 47.25% accuracy.

Researchers proposed various DL-based models for garbage classification, but they often failed to recognize tiny garbage articles and min-overlapped articles. Therefore, a lightweight DL model is needed for the effective detection and classification of garbage particles. Table 1 shows a comparative analysis of the existing DL models.

**Table 1:** Comparative analysis of the existing models over the garbage classification dataset.

Author	Year of Publication	Proposed Model	Accuracy
Paneru et al.^6^	2024	Tkinter-based Recycling using PyTorch	96.32%
Ahmed et al.^10^	2023	DenseNet169	94.40%
		MobileNetV2	97.60%
		ResNet50V2	98.95%
Srilatha et al.^11^	2023	Faster RCNN with ResNet50	93.30%
		Faster RCNN	86.70%
		Faster RCNN -ResNet101	96.70%
Vijayalakshmi et al.^12^	2023	EBMOHDL-WC	98.04%
		AEOIDL-SWM	97.14%
		MLH-CNN	91.94%
		DLSODC-GWM	96.93%
		RestNet50	73.55%
		VGG16	72.46%
		AlexNet	67.39%
Bobde et al.^13^	2023	EfficientNet-B3 CV model	97.00%
Tatke et al.^14^	2021	Simple CNN	82.19%
		VGG16	75.60%
		ResNet50	95.93%
		HOG + SVM	47.25%

### Deep learning

Deep learning is a field of ML that changed the world with its ability to learn from huge datasets based on hidden patterns. DL is used in many areas, such as fraud detection, algorithmic trades, and estimating risks in deep learning. Other applications include entertainment, such as recommendation systems, robotics, and cybersecurity. Deep learning automatically extracts meaningful features from images to identify subtle patterns and anomalies that might otherwise go unnoticed by the human eye. There are various applications of deep learning, showcasing its potential to revolutionize fields like image analysis, Natural language processing (NLP), Data analysis, healthcare, and autonomous vehicles. Deep learning models use complex mathematical calculations to understand the association between input image attributes and target variables. Classification can be described as a supervised learning category within the area of machine learning, whereby an algorithm predicts a class or categorical label to which any instance would belong based on its features. Classification involves the training of a model on a training dataset that is labeled whereby, for every instance, there is a set of features along with a target variable. The learned features from the image can be used to predict the target variable. After training this model, it can predict class labels on unseen and new instances. Classification is a diverse technique of machine learning that predicts categorical labels or classes based on given features. Its applications are versatile and have been put into use widely.

### CNN

Deep learning architectures, mainly CNNs, have a significant impact on the field of digital image processing. CNN is primarily used to perform image processing-related tasks like object detection, classification, and object recognition. It is designed with multi-layered convolutional (conv) layers to extract the essential features from the images. CNN uses a series of convolutional layers to learn various hidden features. The pooling layers are used to reduce the dimensionality, and the fully connected layers are used to produce the final outputs. CNN works directly on the images instead of focusing on extracting features as other neural networks do.

### Transformer

Transformers have revived deep learning, specifically natural language processing. Traditionally, the mode that makes use of a recurrent neural network (RNN) cannot weigh the input elements well since transformers are powered off a self-attention (SA) mechanism. Therefore, they are better able to capture the long-range dependencies, which makes the transformers perform better on tasks such as machine translation (MT), text summarization, and so forth^15^. Parallel instead of sequential processing dramatically improved the efficiency and scalability of such models. Most of the transformer architecture is the basis of many state-of-the-art NLP systems whose scope of applications remains unlimited within the currently identified domains. The Transformer architecture^16^ comprises an Encoder and a Decoder, each consisting of stacked modules (Nx). These modules mainly incorporate Multi-Head Attention and Feed-forward layers. Input and target sentences are initially embedded into an n-dimensional space for processing. The Transformer effectively transforms one sequence into another by leveraging the interaction between these modules.

### Vision transformer

**Fig. 2:**
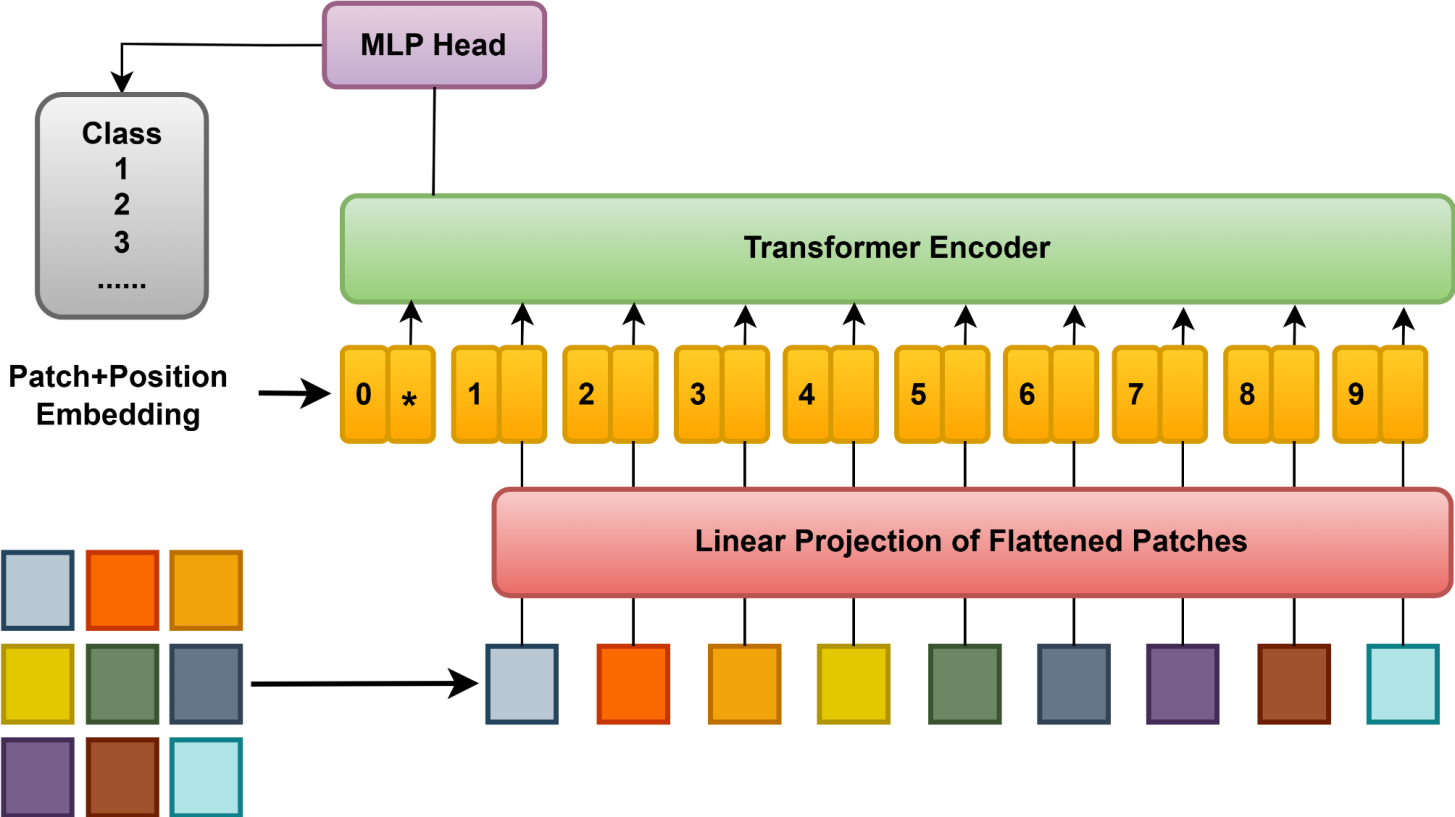
Working of vision transformer.

Vision Transformers (ViT)^17,18^, as a new paradigm in computer vision, proved to be a promising new alternative for recognizing and understanding images. Unlike traditional convolutional neural networks, the attention mechanism is used in NLP to process the images as a sequence of patches. This enables an end to capture long-range dependencies in images and can, ultimately, give superior performance in object detection, image classification, and semantic segmentation. The ViT (Fig. 2) is utilized as a Transformer-like image patch architecture, dividing the image into fixed-sized patches. Then, each of these blocks is embedded linearly, position embeddings are added, and the result of the vectors is fed into the standard Transformer encoder. The standard method of combining additional learning “classification symbols” to the series is used to do classification.

### Swin transformer

**Fig. 3:**
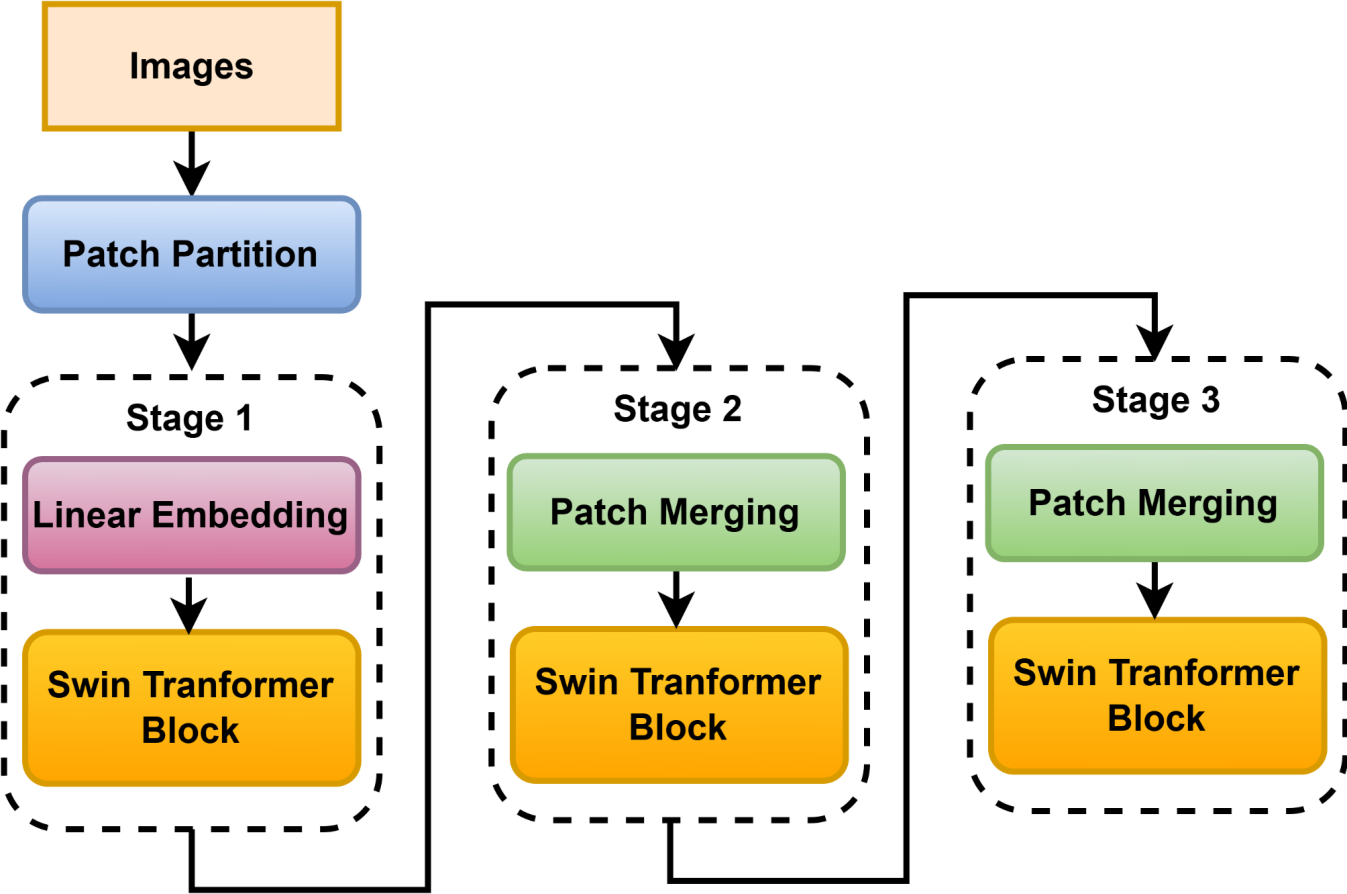
Swin transformer architecture.

Swin Transformer^19,20^ is an advanced deep-learning model based on transformers, showing outstanding performance in computer vision-related tasks and surpassing most of the CNN and transformer models. Employing a hierarchical structure with shifted window attention can computationally efficiently capture long-range dependencies. This enables the model to handle large diversity in visual scales and obtain best-in-class results for image processing tasks. Image classification using a Swin Transformer (Fig. 3) follows a certain sequential number of steps. Firstly, the input image is divided into a set of fixed-size patches. Then, sequentially projected bedding vectors are created by multiplying them with a learnable weight matrix. It then passes through stages of the Swin Transformer sequentially at every patch within a series of shifted convolutions and hierarchical self-attention mechanisms.

### ConvNeXt

**Fig. 4:**
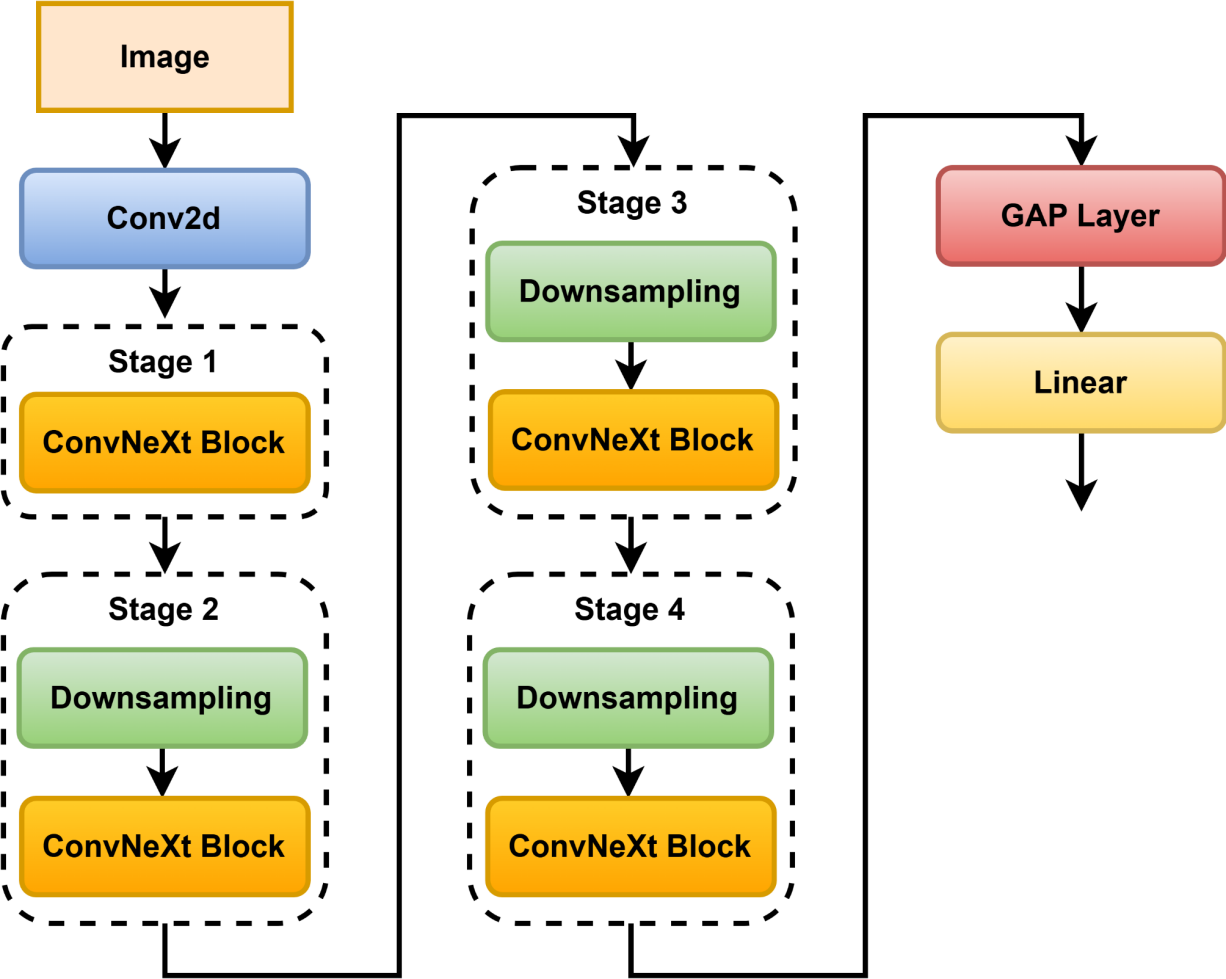
ConvNeXt architecture.

The architecture of ConvNeXt^21^ comprises a set of ConvNeXt Blocks, each containing a Convolutional Layer. This layer uses a large kernel size, such as 7 × 7, to capture long-range dependencies. Layer Normalization normalizes the output of the convolutional (conv) layer. Depth-wise convolution reduces both the total number of parameters and computational cost. The Gaussian Error Linear Unit (GELU)^22^ is used as an activation function; it weights the input by its probability under a Gaussian distribution. It has a hierarchical structure^23^ (Fig. 4) with several stages and various resolutions.

Stage 1: In this stage, the patches are resampled at a resolution of at least 1/4 of the original image.

Stage 2: Down-sampled patches are processed at a lower resolution, e.g., half the size of the original image.

Stage 3: Down-sampled patches are processed at reduced resolution, for instance, 3/4 of the original size of the image.

Stage 4: Repeat the process until the last stage.

Down-sampling uses stridden convolution to down-sample patches between stages. Up-sampling uses either bilinear interpolation or transposed convolution to up-sample patches in the final stage. It generates a final output that can be used for any classification, detection, segmentation, or generation tasks. ConvNeXt combines the best of the CNNs and ViTs with SOTA results on several CV standards.

## Proposed method

**Fig. 5:**
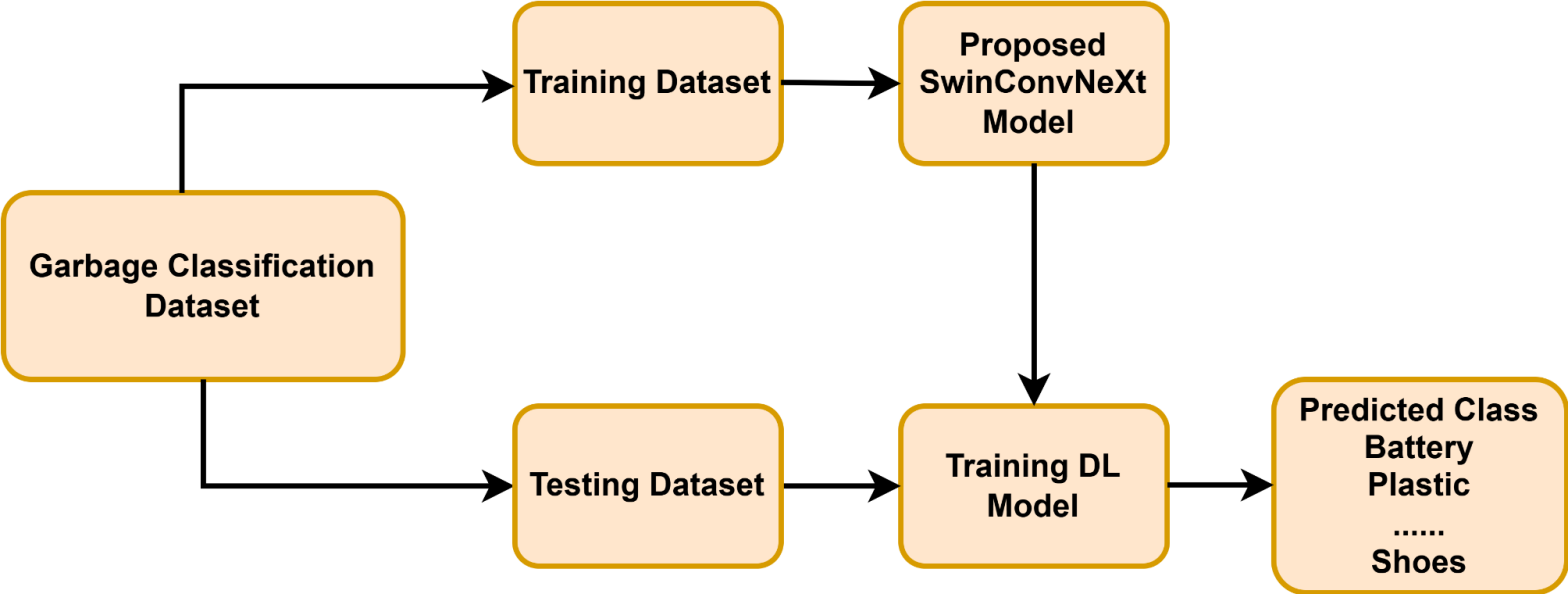
Flow graph for garbage classification using proposed SwinConvNeXt.

Garbage classification in real-time systems is challenging due to the mixed nature, size, and shape of the particles. Therefore, a lightweight DL model is needed for the effective detection and classification of garbage particles. A novel Fusion Deep Learning architecture, integrating the strengths of Swin Transformer and ConvNext, is proposed for robust garbage classification. The enhanced Swin Transformer, with its global multi-head attention layer, excels at capturing long-range dependencies and global context within the image. This is crucial for understanding the spatial relationships between different objects and their overall distribution within the scene. The improved ConvNeXt block, with its efficient convolutional operations, excels at extracting local features and fine-grained details. This is essential for accurately identifying and characterizing individual garbage particles within the image. The flow graph for the proposed SwinConvNeXt model is depicted in Fig. 5.

The proposed model is developed by stacking the Swin Transformer and ConvNeXt deep learning model. The fused deep learning model demonstrates significant advantages over individual models by effectively leveraging the complementary strengths of the Enhanced Swin Transformer and improved ConvNeXt Deep Learning models.

- Complementary Strengths: The fusion of deep learning models effectively mitigates each model’s potential weaknesses. The Swin Transformer, while strong in a global context, might struggle with fine-grained details. Conversely, the ConvNeXt, while strong in local features, might miss crucial long-range relationships. By combining their strengths, the fused model can achieve a more balanced and robust performance.
- Enhanced Generalization: The diverse feature representations extracted by the two models improve the model’s ability to generalize to unseen data. This is crucial for real-world applications where the model needs to be able to classify garbage accurately and segment it accurately in various environmental conditions and with varying degrees of clutter.
- Diverse Information Sources: The fused model leverages information from both global and local feature representations. This diversity can make the model more robust to noise, occlusions, and variations in image quality, which are common challenges in real-world garbage classification scenarios.
- Improved Computational Efficiency: The improved ConvNeXt block incorporates modifications such as the Normalized layer and the use of ReLU activation, which can potentially improve computational efficiency compared to the original ConvNeXt architecture.
- Potential for Architectural Optimizations: The fusion process itself can be further optimized by exploring different fusion strategies and architectural designs, potentially leading to more efficient and computationally less expensive models.

**Fig. 6:**
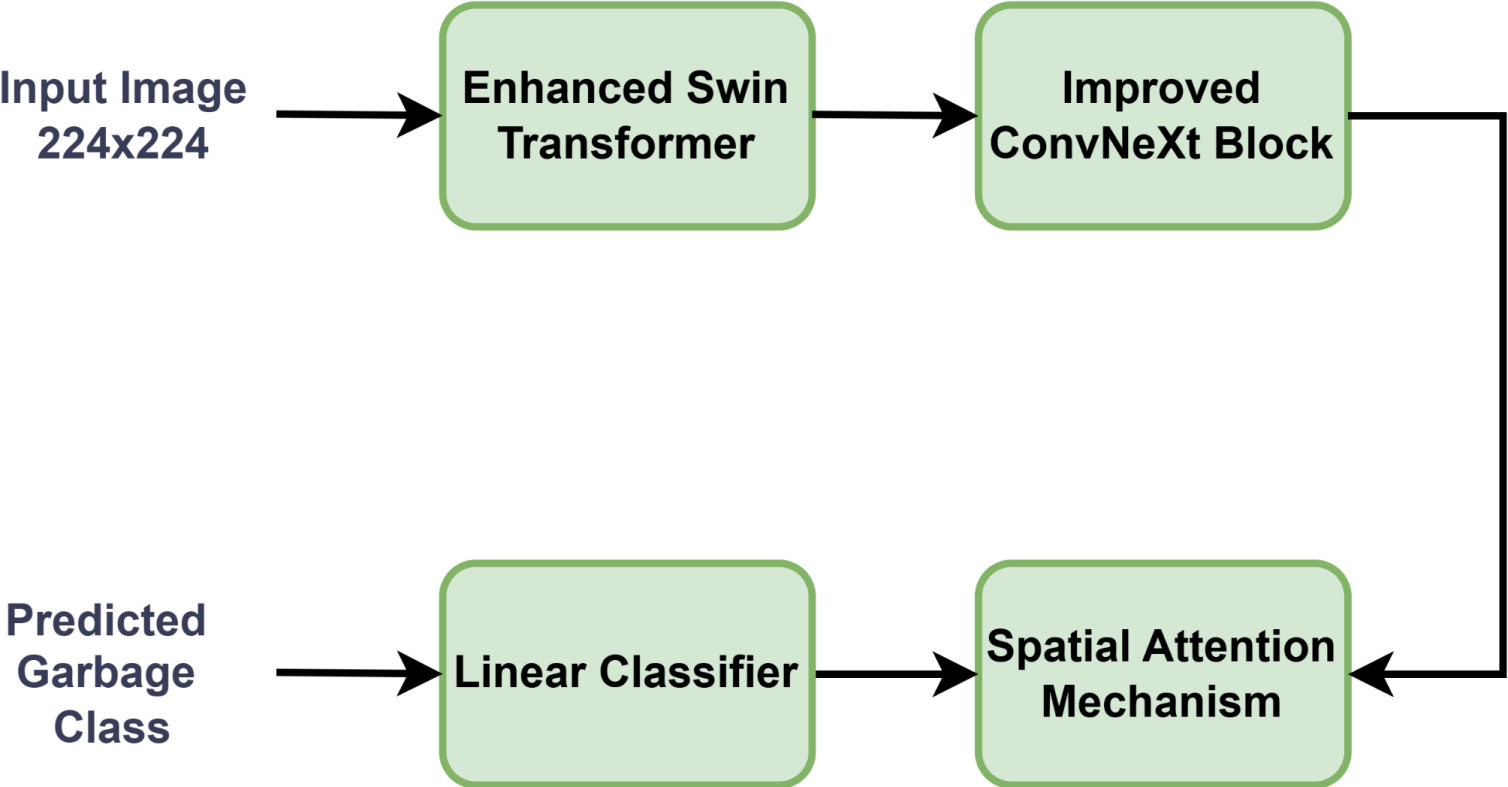
Proposed SwinConvNeXt DL model architecture.

The proposed fused model attains the best accuracy with less computational time and by using only five epochs. The proposed system architecture is shown in Fig. 6.

The proposed fused deep learning model operates sequentially. Firstly, the input image is processed by the enhanced Swin Transformer, which employs a hierarchical architecture with shifting window multi-head self-attention to extract discriminative global features. Subsequently, the improved ConvNeXt block processes the input image to capture fine-grained local features. The extracted features from both pathways are then fused at a designated layer. Finally, the fused features are fed into a fully connected layer followed by a SoftMax activation function for classification, resulting in the predicted class probabilities. The working algorithm of the proposed model is as follows:

**Algorithm:**
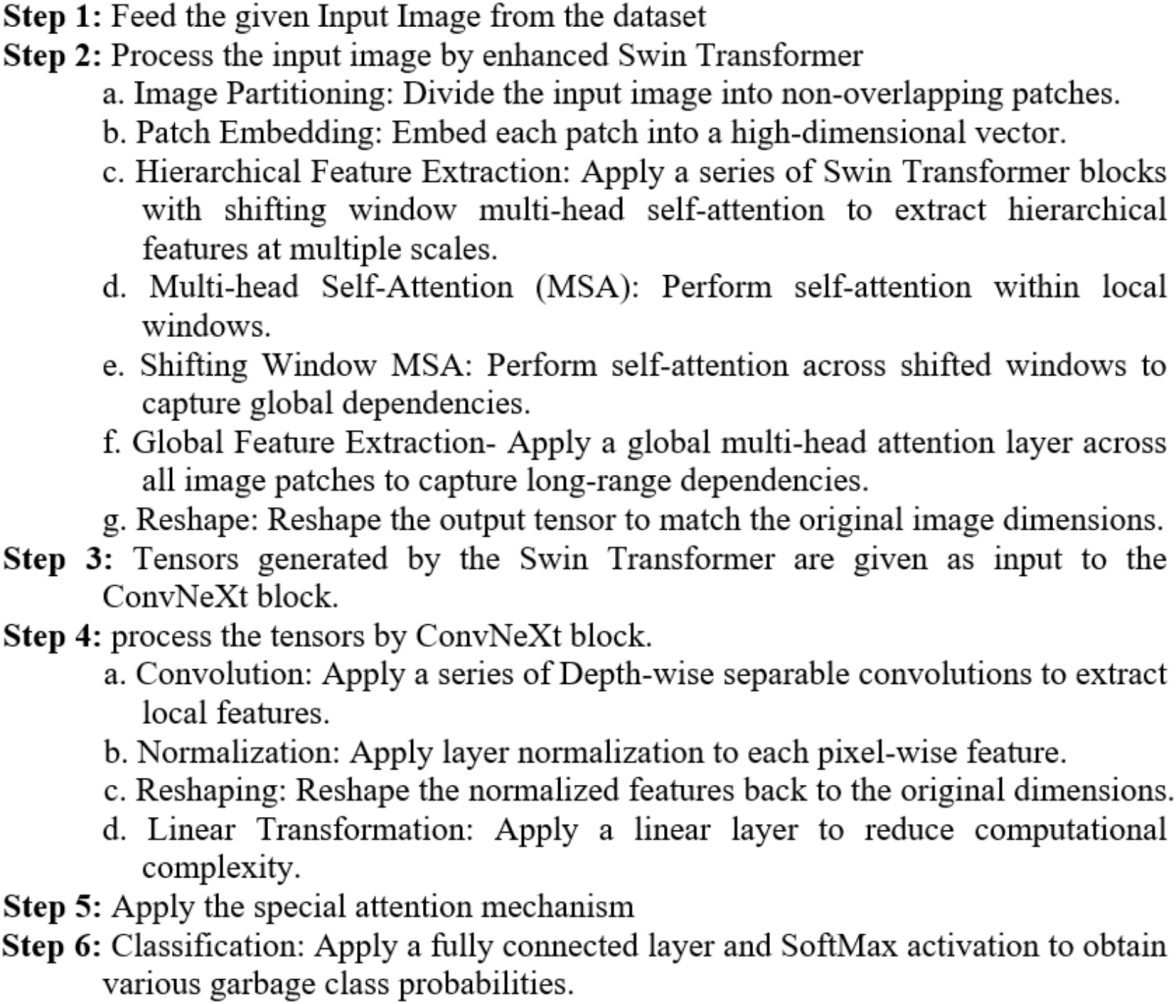
Fused SwinConvNeXt deep learning model.

### Enhanced swin transformer

**Fig. 7:**
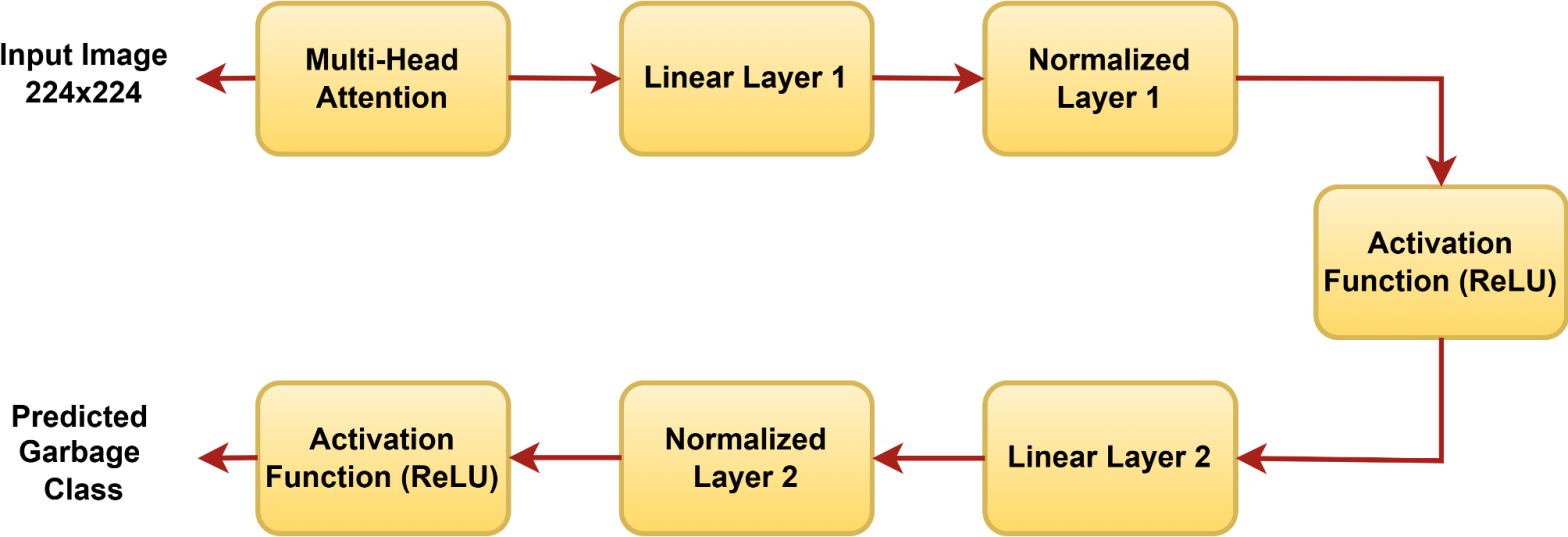
Enhanced swin transformer.

Swin Transformer is a robust vision transformer architecture that performs well for several tasks in computer vision. In the proposed system, we enhanced the Swin Transformer for more effective feature extraction. The enhanced Swin Transformer can acquire the maximum possible global features from the given input image. As shown in Fig. 7, a multi-head attention layer is attached to the existing Swin transformer.

The incorporation of the shifting window mechanism within the Swin Transformer component significantly enhances the efficiency of our proposed fused deep learning model. This mechanism effectively reduces computational complexity by partitioning the image into smaller overlapping windows, enabling efficient parallel processing. Furthermore, the hierarchical structure of the Swin Transformer allows for efficient feature extraction at multiple scales, capturing both fine-grained details and global context with minimal computational overhead.

The input image (224 × 224 × 3) is fed into the Swin transformer, and then the multi-head attention layer applies an attention mechanism across the image patches. The multi-head attention layer expects an input of shape (S, N, E), where S is sequence length (i.e., a number of patches or flattened dimensions), N is the batch size, and E is the embedding dimension (3 channels here). The output from the attention layer will have the same shape as the input (1, 50176, 3). The output is transformed by two linear layers, linear layer1 and linear layer2. Layer Normalizations are applied after each linear transformation layer to stabilize the training process, followed by the ReLU activation function^25^. Finally, the tensor is reshaped back to the image dimensions.

We used the Multi-Head Attention layer, which accomplishes global attention rather than window-based attention. It is like Shifted Window Multi-Head Self-Attention (SW-MSA), which allows the capture of the dependencies over the entire image. Our enhanced Swin Transformer uses the ReLU activation function, whereas the basic Swin Transformer uses GELU. We used reshaping and linear layers to adjust the dimensions before and after attention. The basic Swin Transformer used MLP (Multi-Layer Perceptron) layers and normalization in each stage. The computational complexity of multi-head self-attention (MSA) and window multi-head self-attention (W-MSA) are presented in Eqs. (1) and (2), respectively. In Eq. (1), MSA’s complexity is quadratic with respect to the product of height (h) and width (w).

1 $${\text{\varvec{\Omega}MSA=4hw}}{{\text{C}}^{\text{2}}}{\text{+2(hw}}{{\text{)}}^{\text{2}}}{\text{C}}$$

2 $${\text{\varvec{\Omega}W-MSA=4hw}}{{\text{C}}^{\text{2}}}{\text{+2}}{{\text{M}}^{\text{2}}}{\text{hwC}}$$

Conversely, in Eq. (2), W-MSA’s complexity exhibits a linear relationship with the product of h and w when the window size (M) is fixed. Where C denotes the dimension, and h and w are the height and width of the image, respectively. This hierarchical approach not only improves efficiency but also contributes to the model’s overall accuracy and robustness.

### Improved ConvNeXt block

**Fig. 8:**
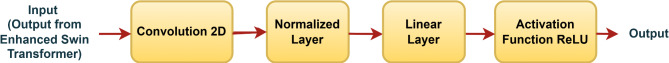
Improved ConvNeXt Block.

The Improved ConvNeXt Block is inspired by the base ConvNeXt neural network architecture, a type of CNN designed for image classification tasks. The improved ConvNext block captures most of the local features, which helps provide fine details of every garbage particle. The improved ConvNeXt block is shown in Fig. 8.

The ConvNeXt block receives the original image tensor (1, 3, 224, 224). The convolution operation applies to the image, which keeps the spatial dimensions the same but increases the number of channels. Then, the output shape after convolution is (1, 64, 224, 224). The tensor is permuted and reshaped for normalization. Here, normalization is applied to each pixel-wise feature, which is then reshaped back and followed by the ReLU activation function. We used the Normalized layer, which is used for normalizing layers on flattened data, instead of the more typical normalization directly applied post-convolution. After the normalization layer, we introduced a linear layer to reduce computations. We used the ReLU activation function instead of GELU for its computational efficiency. ReLU’s sparse activations can help in regularization and better feature selection.

### Spatial attention mechanism

The spatial attention mechanism is often used in computer vision (CV) tasks, such as image captioning, visual question answering, and object detection, to focus on certain regions of the input image selectively. The spatial attention mechanism applies spatial attention to the feature map, highlighting important regions. It consists of a Conv2d layer that reduces the channel dimension from 128 to 1 by creating a spatial attention map. The number of input channels that the convolutional layer will accept is 128. The number of output channels that the convolutional layer will produce is 1, which means the output will be a single feature map. A kernel size of 1 means that the filter will be 1 × 1, which is equivalent to a dot product between the input and the filter weights. By applying a 1 × 1 convolution, attention maps that highlight significant areas of the feature maps are generated. This filter reduces the number of channels and retains spatial information.

**Fig. 9:**
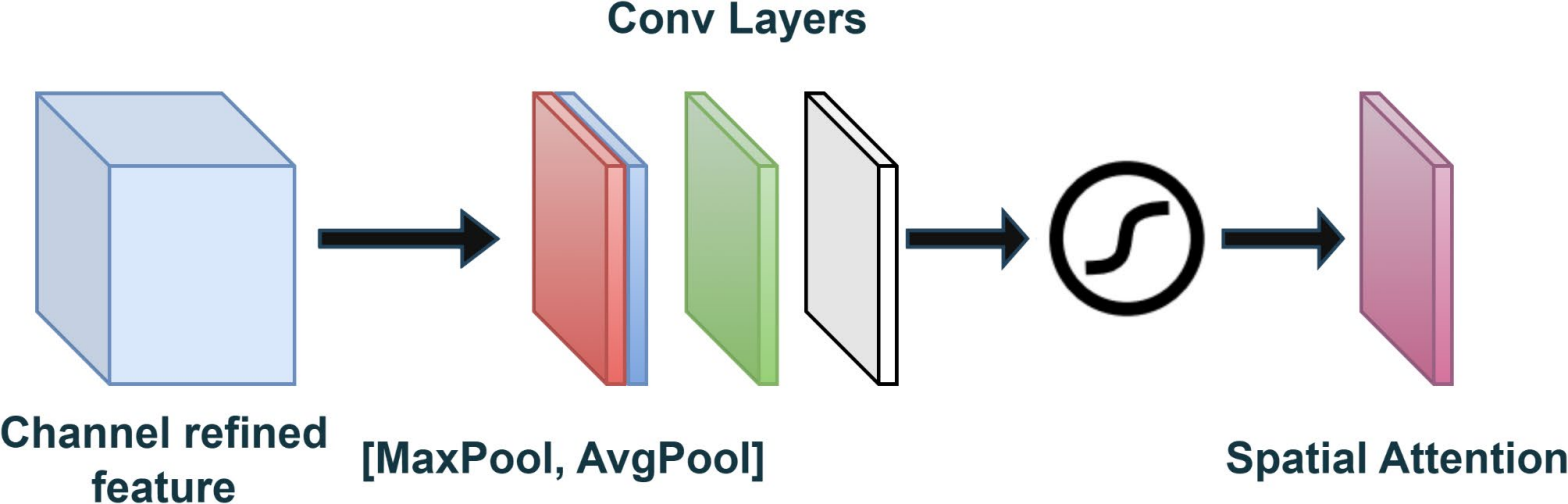
Spatial attention module.

Figure 9 shows the Spatial Attention Module. We aggregated the channel information from a feature map using average and max pooling, resulting in two 2D maps. Subsequently, these maps are concatenated and convolved using a standard convolution layer to generate the 2D spatial attention map.

### Linear classifier

This work uses a classifier using a linear neural network layer to attain better accuracies in image classification tasks. The number of input features that the linear layer will accept is 224 ×  224, so the input to the classifier is an image with a size of 224 × 224 pixels, flattened into a 1D array. The total number of output features that the linear layer will produce is 12, which likely indicates the total number of classes in the garbage classification problem.

## Experimental results and analysis

### Dataset

The classification of household garbage into more granular categories can significantly enhance the efficiency of the recycling process and boost the overall percentage of recycled waste. We used the publicly available benchmark Garbage collection Dataset^26^ for our research. The dataset comprises a total of 15,150 images, representing 12 different categories like paper, biological, cardboard, metal, green glass, plastic, white glass, brown glass, batteries, clothes, shoes, and trash. Table 2 provides a detailed overview of these categories in the Garbage collection dataset.

**Table 2:** Dataset categories.

S.No	Category	Number of Images
1	Battery	945
2	Biological	985
3	Brown Glass	607
4	Cardboards	891
5	Clothes	5325
6	Green Glass	629
7	Metal	769
8	Paper	1050
9	Plastic	865
10	Shoes	1977
11	Trash	697
12	White glass	775

### Data pre-processing

Data preprocessing steps are crucial for preparing the garbage classification dataset for effective training and evaluation of the deep learning model. We Resized the Image to 224 × 224, which ensures uniform input for the model and improves computational efficiency. The dataset is pre-processed and then divided into training (80%) and test (20%) datasets. The model is trained for five epochs and optimized using Adam optimization^24^ for optimal performance.

### Experimental setup

All the experiments were carried out on a computer with i7 PROCESSOR-12,700 (16 CORE 25 MB CACHE), NVIDIA CHIPSET GEFORCE GTX1630 4 GB GDDR6 GPU, CPU WITH 32GB of RAM. We build the SwinConvNeXt DL model for categorizing different types of waste products using Python 2.7 on Keras and TensorFlow. The training of the proposed DL architecture is done by using the 80% images of the Garbage collection Dataset over five epochs.

### Evaluation metrics

Evaluation metrics are quantifiable measures that indicate how well an analytical or DL model is performing in terms of being effective. Metrics gives insight into how well the model is doing and compares one model to another. In assessing a DL model, prime importance is attached to predictability, generalization capability, quality, and overall ranking. Depending upon the problem domain, the nature of the data, and the possible outcome, the appropriate selection of an evaluation metric is indispensable. We considered the confusion matrix, Accuracy, Precision, Recall, and F- measure, which provides a comprehensive view of the proposed SwinConvNeXt classification model over the garbage classification dataset.

## Results and discussions

The proposed model was evaluated using the standard benchmark Garbage collection dataset. The model performed excellently during the training. We first assessed the proposed enhanced Swin Transformer, which attains an accuracy of 78.23%. When we evaluated the modified ConvNext model, it achieved an accuracy of 80.12%. When we perform hyperparameter tuning to obtain the optimal values for all hyperparameters and get the best possible result. Due to the lightweight nature and low computational power of the proposed SwinConvNeXt, all images were correctly classified with their labels.

**Fig. 10:**
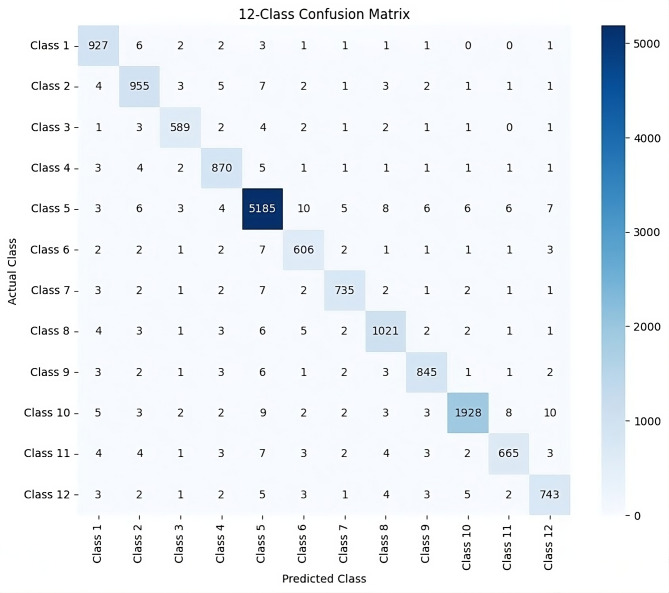
Confusion matrix.

Figure 10 compares the actual values of the garbage classification dataset with the predicted values, allowing us to understand how well the proposed model is classified into different categories. Confusion matrix is a tabular representation that measures the performance of the classification model. It defines the count of true positives, false positives, true negatives, and false negatives. The matrix typically shows a comprehensive evaluation of the proposed model over the test dataset. We compared the obtained results of the proposed model with all the existing SOTA models. Table 3 shows the comparative result analysis of the proposed model with existing models.

**Table 3:** Comparison with baseline models.

Reference	Accuracy	Precision	Recall	F_Score
PyTorch^6^	96.32	97.54	95	96.25
DenseNet169^10^	94.40	94.00	94.00	94.00
MobileNetV2^10^	97.60	95.00	97.00	97.00
ResNet50V2^10^	98.95	98.35	98.38	98.38
Faster RCNN with ResNet50^11^	93.3	96.36	90.00	93.07
Faster RCNN (Simple CNN)^11^	86.70	88.07	85.60	86.82
Faster RCNN -ResNet101^11^	96.70	98.34	95.00	96.64
EfficientNet-B3 CV model^13^	97.00	98.35	95.60	96.96
Simple CNN^14^	82.19	83.68	80.00	81.80
ResNet50^14^	95.93	94.92	93.00	95.03
Proposed Swin Transformer	78.23	77.32	78.54	77.26
Proposed ConvNeXt	80.12	78.12	79.58	79.10
Proposed (SwinConvNeXt) Model	98.97	98.42	98.61	98.56

The proposed SwinConvNeXt model demonstrates superior performance in garbage image classification, achieving notable improvements in key evaluation metrics. Accuracy is probably one of the most widely used metrics for evaluating the performance of a classification model. It measures the percentage of all samples correctly classified out of all samples in total. The accuracy is calculated as shown in Eq. 3.

3 $${\text{Accuracy}} = \frac{{{\text{TP+TN}}}}{{{\text{TP+TN+FP+FN}}}}$$

**Fig. 11:**
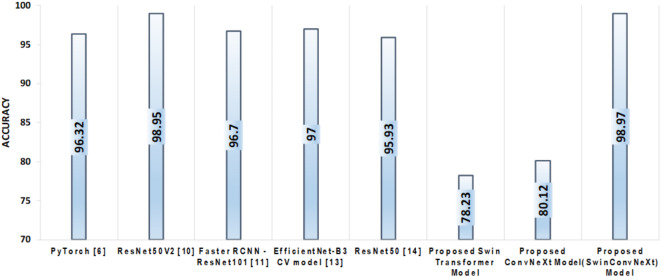
Comparison of SOTA models with proposed models based on Accuracy.

The Enhanced Swin transformer model attains 78.23% accuracy, while the enhanced ConvNeXt model attains 80.12% accuracy. The proposed SwinConvNeXt model achieves a better classification accuracy of 98.97%, surpassing the existing garbage classification models, as shown in Fig. 11.

Precision, a crucial metric for evaluating classification performance, measures the proportion of true positive predictions among all positive predictions, as shown in Eq. 4.

4 $${\text{Precision}} = \frac{{{\text{TP}}}}{{{\text{TP+FP}}}}$$

**Fig. 12:**
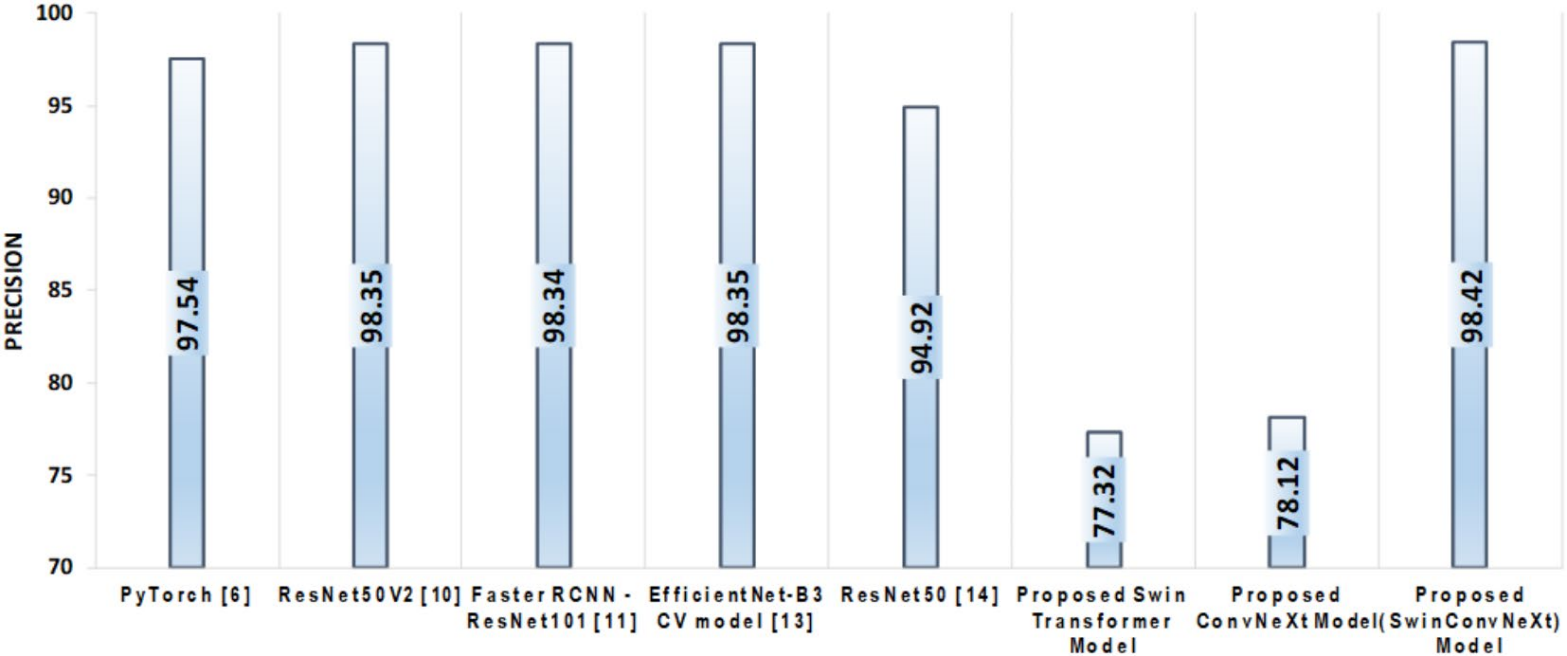
Comparison of SOTA models with proposed models based on Precision.

In our experiments, the Enhanced Swin Transformer model achieved a precision of 77.32%, while the improved ConvNeXt model attained 78.12%. Notably, the proposed SwinConvNeXt model significantly outperformed both individual models, achieving a precision of 98.42%. This substantial improvement in precision demonstrates the effectiveness of the proposed architecture in accurately identifying garbage types, minimizing the number of false positive classifications, and thereby enhancing the reliability of the overall waste management system, as shown in Fig. 12.

Recall, a crucial metric in classification, assesses the model’s ability to correctly identify all actual positive instances. As defined in Eq. 5, recall is calculated as the ratio of true positives to the total number of actual positive samples.

5 $${\text{Recall}} = \frac{{{\text{TP}}}}{{{\text{TP+FN}}}}$$

**Fig. 13:**
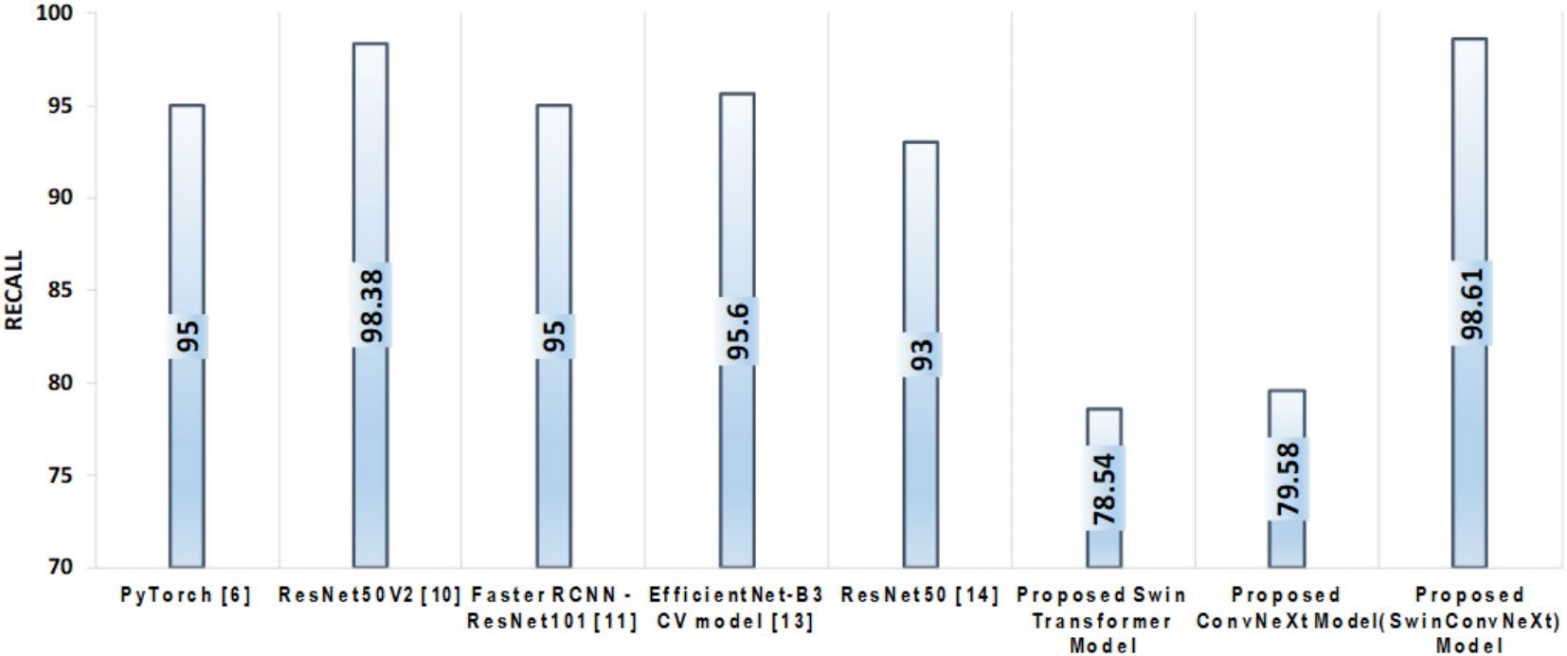
Comparison of SOTA models with proposed models based on Recall.

In our experiments, the Enhanced Swin Transformer model achieved a recall of 78.54%, while the Enhanced ConvNeXt model attained 79.58%. Notably, the proposed SwinConvNeXt model significantly outperformed both individual models, achieving a recall of 98.61%, as depicted in Fig. 13.

The F-measure provides a balanced assessment of precision and recall by calculating their harmonic mean, as defined in Eq. 6. This metric is particularly valuable as it considers the model’s ability to minimize false positives (precision) and its ability to correctly identify all relevant instances (recall).

6 $$F - Measure=\frac{{2*\operatorname{Re} call*\operatorname{Precision} }}{{\operatorname{Re} call+\operatorname{Precision} }}$$

**Fig. 14:**
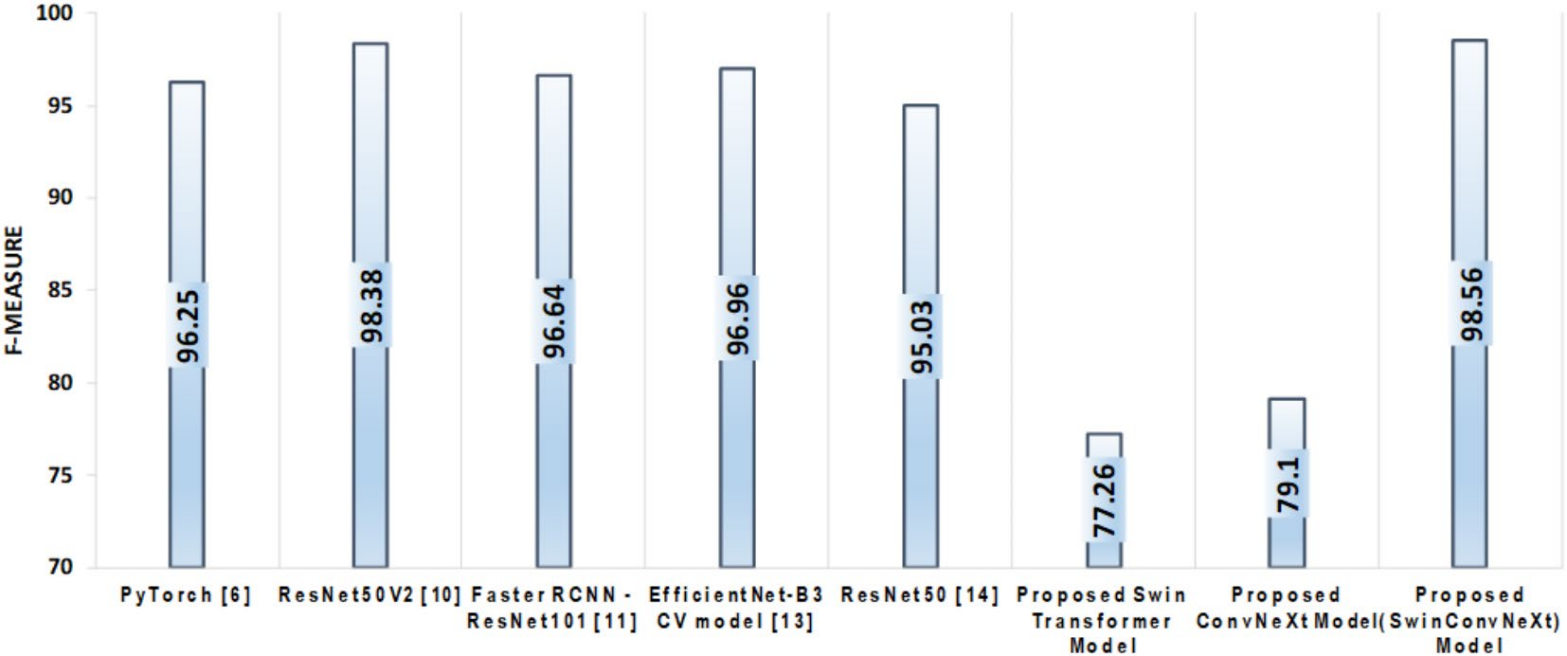
Comparison of SOTA models with proposed models based on F-measure.

In our experiments, the Enhanced Swin Transformer model achieved an F-Measure of 77.26%, while the Enhanced ConvNeXt model attained 79.1%. Notably, the proposed SwinConvNeXt model significantly outperformed both individual models, achieving an F-Measure of 98.56%, as depicted in Fig. 14.

Along with the existing SOTA models, recent studies^5,7,8^ have explored deep learning models for garbage classification with notable successes. Building upon these advancements, our proposed SwinConvNeXt model significantly outperforms these previous approaches. This substantial improvement, coupled with the model’s lightweight architecture and resource efficiency, underscores the efficacy of SwinConvNeXt in real-time garbage image classification tasks. This paves the way for more robust and reliable waste management systems, particularly in resource-constrained environments where computational power and memory are limited.

## Conclusion and future scope

This research paper introduces SwinConvNeXt, a novel deep-learning architecture that effectively tackles the challenges of real-time garbage image classification. By synergistically combining the strengths of an enhanced Swin Transformer, improved ConvNeXt, and a spatial attention mechanism, the proposed model achieves exceptional performance, attaining 98.97% accuracy, 98.42% precision, and 98.61% recall on the benchmark Garbage Classification dataset. The integration of the enhanced Swin Transformer’s shifting window mechanism and hierarchical feature extraction enables efficient capture of long-range dependencies within the image, which is crucial for distinguishing visually similar garbage types. Concurrently, the improved ConvNeXt block excels at extracting fine-grained local features, which is crucial for identifying subtle variations in shape, texture, and appearance. The lightweight nature and low computational cost of SwinConvNeXt make it well-suited for real-time deployment in resource-constrained environments, such as on-device applications for smart waste management systems. Future research directions include investigating real-time object detection and tracking algorithms in conjunction with SwinConvNeXt for more robust and practical garbage classification systems, with a focus on edge device deployment for on-site waste management. Multi-modal integration with sensors like RGB-D cameras, hyperspectral imaging, and even acoustic sensors can further enhance accuracy and robustness. Utilizing 3D images instead of 2D images can effectively address the challenges posed by overlapped objects, leading to more accurate waste sorting. Additionally, enhancing model robustness and generalization can be achieved through advanced data augmentation techniques like style transfer, adversarial training, and synthetic data generation. Finally, exploring mechanisms for dynamic model adaptation based on the specific waste stream characteristics, environmental conditions, and user feedback will enhance efficiency, accuracy, and adaptability in diverse real-world scenarios.

## Electronic supplementary material

Below is the link to the electronic supplementary material.

Supplementary Material 1

## Data Availability

The dataset utilized in this work is freely available: https://www.kaggle.com/datasets/mostafaabla/garbage-classification.
