# Supplementary material for: SwinConvNeXt: a fused deep learning architecture for Real-time garbage image classification
Source: Sci Rep. 2025 Mar 7;15:7995. doi: 10.1038/s41598-025-91302-7 (PMC11889143; doi:10.1038/s41598-025-91302-7)
Supplement: Supplementary file 1 — Supplementary Material 1 [file 41598_2025_91302_MOESM1_ESM.docx]

**SwinConvNeXt: A Fused Deep Learning Architecture for Real-time Garbage Image Classification**

**Algorithm:** Fused SwinConvNeXt Deep Learning Model

**Step 1:** Feed the given Input Image from the dataset

**Step 2:** Process the input image by enhanced Swin Transformer

a. Image Partitioning: Divide the input image into non-overlapping patches.

b. Patch Embedding: Embed each patch into a high-dimensional vector.

c. Hierarchical Feature Extraction: Apply a series of Swin Transformer blocks with shifting window multi-head self-attention to extract hierarchical features at multiple scales.

d. Multi-head Self-Attention (MSA): Perform self-attention within local windows.

e. Shifting Window MSA: Perform self-attention across shifted windows to capture global dependencies.

f. Global Feature Extraction- Apply a global multi-head attention layer across all image patches to capture long-range dependencies.

g. Reshape: Reshape the output tensor to match the original image dimensions.

**Step 3:** Tensors generated by the Swin Transformer given as input to the ConvNeXt block.

**Step 4:** process the tensors by ConvNeXt block.

a. Convolution: Apply a series of Depth-wise separable convolutions to extract local features.

b. Normalization: Apply layer normalization to each pixel-wise feature.

c. Reshaping: Reshape the normalized features back to the original dimensions.

d. Linear Transformation: Apply a linear layer to reduce computational complexity.

**Step 5:** Apply the special attention mechanism

**Step 6:** Classification: Apply a fully connected layer and SoftMax activation to obtain various garbage class probabilities.
